# Supplementary material for: Optimizing the Antimicrobial Activity of Sodium Hypochlorite (NaClO) over Exposure Time for the Control of Salmonella spp. In Vitro
Source: Antibiotics (Basel). 2024 Jan 10;13(1):68. doi: 10.3390/antibiotics13010068 (PMC10812646; doi:10.3390/antibiotics13010068)
Supplement: Supplementary file 1 [file antibiotics-13-00068-s001.zip › antibiotics-2788523-supplementary.pdf]

Supplementary Material

Table S1: Analysis of variance (ANOVA) of *Salmonella* Enteritidis ATCC 13076. This strain was not influenced by exposure time or temperature. NaClO concentration was the only variable significantly affected, with $p<0.05$. Results indicated in red.

Table S1: ANOVA; Var.: *Salmonella* Enteritidis; R-sqr=,91688; Adj:,82337 (2**(3) central composite, nc=8 ns=6 n0=2 Pontos=16 (Spreadsheet7) in Workbook1)

3 factors, 1 Blocks, 18 runs, MS Pure error = ,1519485

DV: *Salmonella* Enteritidis ATCC 13076

Factor	SS	df	MS	F	*p*
(1) °C (L)	0.62571	1	0.62571	4.1179	0.13544
°C (Q)	0.05686	1	0.05686	0.3742	0.583965
(2) NaClO (L)	36.38508	1	36.38508	239.4566	0.000586
NaClO (Q)	6.10283	1	6.10283	40.1638	0.007945
(3) Time (L)	0.60396	1	0.60396	3.9748	0.140182
Time (Q)	0.42898	1	0.42898	2.8232	0.191504
1L by 2L	0.01417	1	0.01417	0.0933	0.780013
1L by 3L	1.19847	1	1.19847	7.8874	0.067379
2L by 3L	0.63661	1	0.63661	4.1896	0.133166
Lack of fit	3.73634	5	0.74727	4.9179	0.110064
Pure error	0.45585	3	0.15195		
**Total SS**	50.4342	17			

Table S2: Analysis of variance (ANOVA) of *Salmonella* Schwarzengrund. This strain was influenced by NaClO concentration and exposure time, presenting $p<0.05$. Results indicated in red.

Table S2: ANOVA; Var.: *Salmonella* Schwarzengrund; R-sqr=,92448; Adj:,83952 (2**(3) central composite, nc=8 ns=6 n0=2 Pontos=16 (Spreadsheet7) in Workbook1)

3 factors, 1 Blocks, 18 Runs, MS Pure error = ,4504172

DV: *Salmonella* Schwarzengrund

Factor	SS	df	MS	F	*p*
(1) °C (L)	0.7816	1	0.7816	1.73529	0.279313
°C (Q)	0.28738	1	0.28738	0.63802	0.482817
(2) NaClO (L)	40.39918	1	40.39918	89.6928	0.002496
NaClO (Q)	4.13074	1	4.13074	9.17093	0.056384
(3) Time (L)	5.14897	1	5.14867	11.43089	0.043061
Time (Q)	1.8899	1	1.8899	4.1959	0.13297
1L by 2L	0.0453	1	0.0453	0.10058	0.771926
1L by 3L	2.49269	1	2.49269	5.53418	0.10008
2L by 3L	0.90071	1	0.90071	1.99973	0.252241
Lack of fit	3.34443	5	0.66889	1.48504	0.395645

Pure error	1.35125	3	0.45042
**Total SS**	62.17682	17	
